# Supplementary material for: Competent immune responses to SARS-CoV-2 variants in older adults following two doses of mRNA vaccination
Source: Nat Commun. 2022 May 24;13:2891. doi: 10.1038/s41467-022-30617-9 (PMC9130515; doi:10.1038/s41467-022-30617-9)
Supplement: Supplementary file 1 — Supplementary Information [file 41467_2022_30617_MOESM1_ESM.pdf]

Supplemental Information

Competent immune responses to SARS-CoV-2 variants in older adults following two doses of mRNA vaccination

Mladen Jergović*[1,2], Jennifer L. Uhrlaub*[1,2], Makiko Watanabe[1,2], Christine M. Bradshaw[1,2], Lisa M. White[3], Bonnie J. LaFleur[3], Taylor Edwards[4], Ryan Sprissler[4,5], Michael Worobey[6], Deepta Bhattacharya[1,3], Janko Nikolich-Žugich[1,2,3,#]

1- Department of Immunobiology, University of Arizona College of Medicine, Tucson, AZ, USA

2- University of Arizona Center on Aging, University of Arizona, College of Medicine, Tucson, Tucson, AZ, USA;

3- BIO5 Institute, University of Arizona, Tucson, USA

4- University of Arizona Genetics Core, University of Arizona, Tucson, AZ, USA

5- Center for Applied Genetics and Genomic Medicine, University of Arizona, Tucson, AZ, USA

6-Department of Ecology and Evolutionary Biology, University of Arizona, Tucson, AZ 85721, USA.

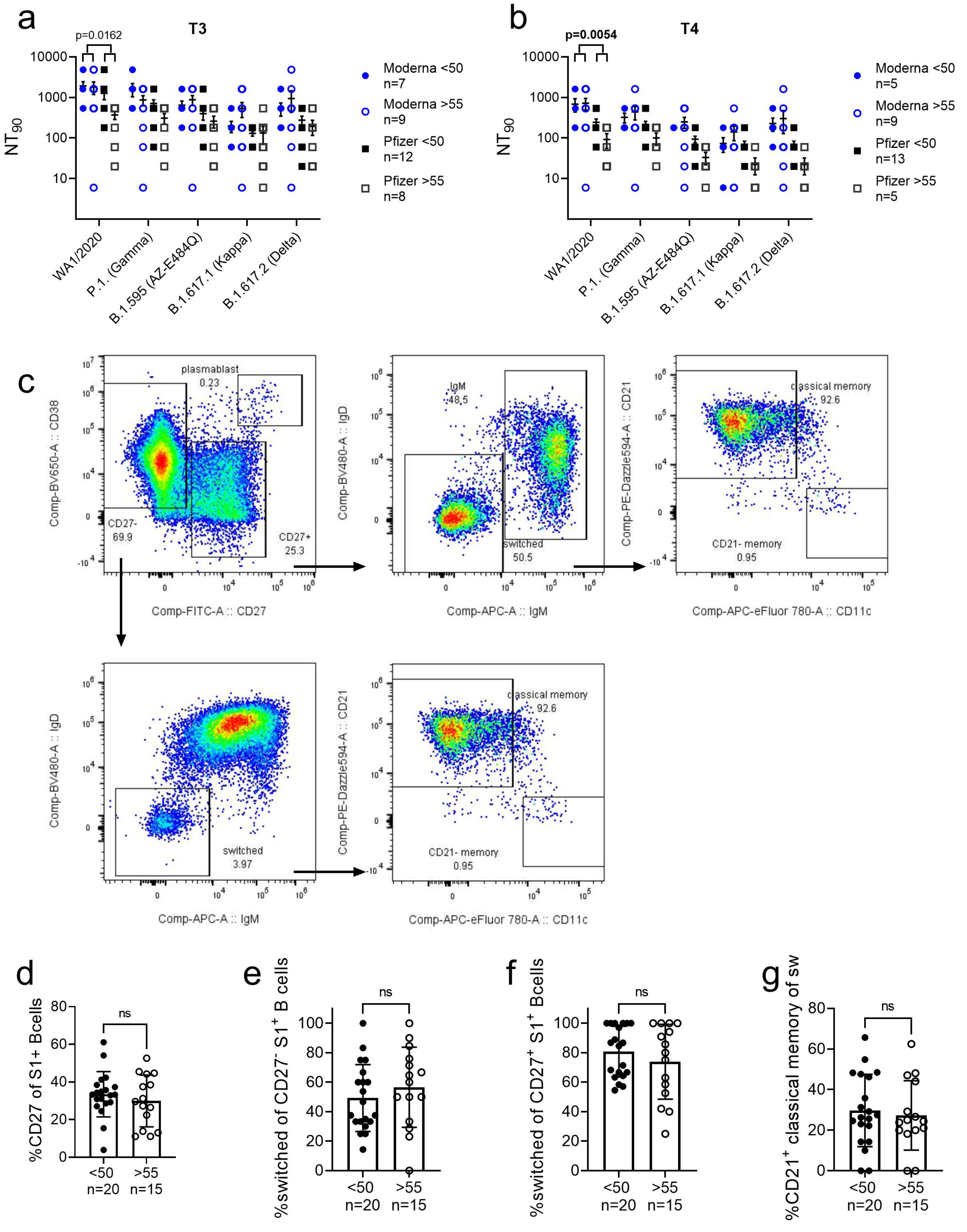
a
T3
p=0.0162
NT90
Moderna <50 n=7
Moderna >55 n=9
Pfizer <50 n=12
Pfizer >55 n=8
WA1/2020
P.1. (Gamma)
B.1.595 (AZ-E484Q)
B.1.617.1 (Kappa)
B.1.617.2 (Delta)
b
T4
p=0.0054
Moderna <50 n=5
Moderna >55 n=9
Pfizer <50 n=13
Pfizer >55 n=5
c
plasmablast 0.23
CD27- 69.9
CD27+ 25.3
Comp-BV650-A :: CD38
Comp-FITC-A :: CD27
IgM 48.5
switched 50.5
Comp-BV480-A :: IgD
Comp-APC-A :: IgM
classical memory 92.6
CD21- memory 0.95
Comp-PE-Dazzle594-A :: CD21
Comp-APC-eFluor 780-A :: CD11c
switched 3.97
d
%CD27 of S1+ Bcells
ns
<50 n=20
>55 n=15
e
%switched of CD27⁻ S1⁺ B cells
f
%switched of CD27⁺ S1⁺ Bcells
g
%CD21⁺ classical memory of sw

Supplementary Fig 1. SARS-CoV-2 specific memory B cells differentiate equally in <50 and >55 cohorts.

Lower neutralization titers in recipients of Pfizer's mRNA vaccine at T3 (**a**) and T4 (**b**)**. c**) B cells gated as in Figure 3A and then as illustrated to examine B cell differentiation of tetramer positive gates. **d**) Percentage of CD27 expressing B cells among tetramer positive cells was equal between cohorts. **e & f**) Equal percentage of SARS-CoV-2 specific B cells were class switched among CD27 positive and negative cells between <50 and >55 cohorts. **g**) Expression of CD21 (Classical memory) was also equal between <50 and >55 cohorts. All data presented as mean values +/- SEM. Two-tailed Mann-Whitney U test.

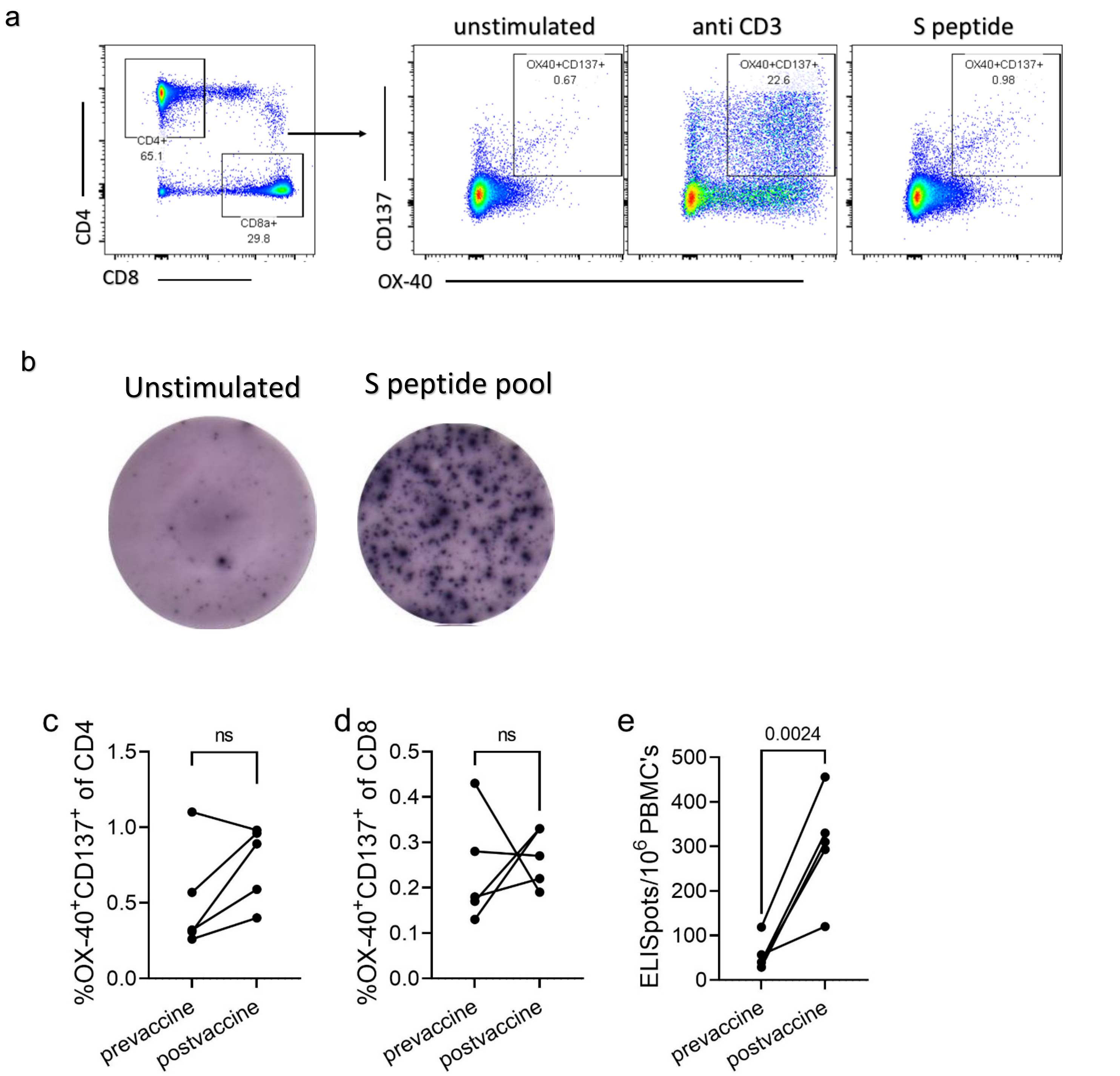

Supplementary Fig 2. ELISpot is more specific method for enumeration of SARS-CoV-2 specific T cells. **a**) Representative flow cytometric gating for identifying CD137+OX-40+ double positive CD4 and CD8 T cells. Singlet live cells were previously gated as in Figure 3A. **b**) Representative unstimulated and Spike protein peptide pool stimulated ELISpot wells. PBMCS from 5 participants were stimulated overnight with USA-WA1/2020 spike peptide pool. SARS-CoV-2 specific T cells were enumerated by either flow cytometry (**c,d**) or ELISpot (**e**). ELISpot proved to be a more sensitive method with statistically significant difference before and after vaccination with N=5. All data presented as mean values +/- SEM. Two-tailed Mann-Whitney U test, for all statistical differences **p<0.01.

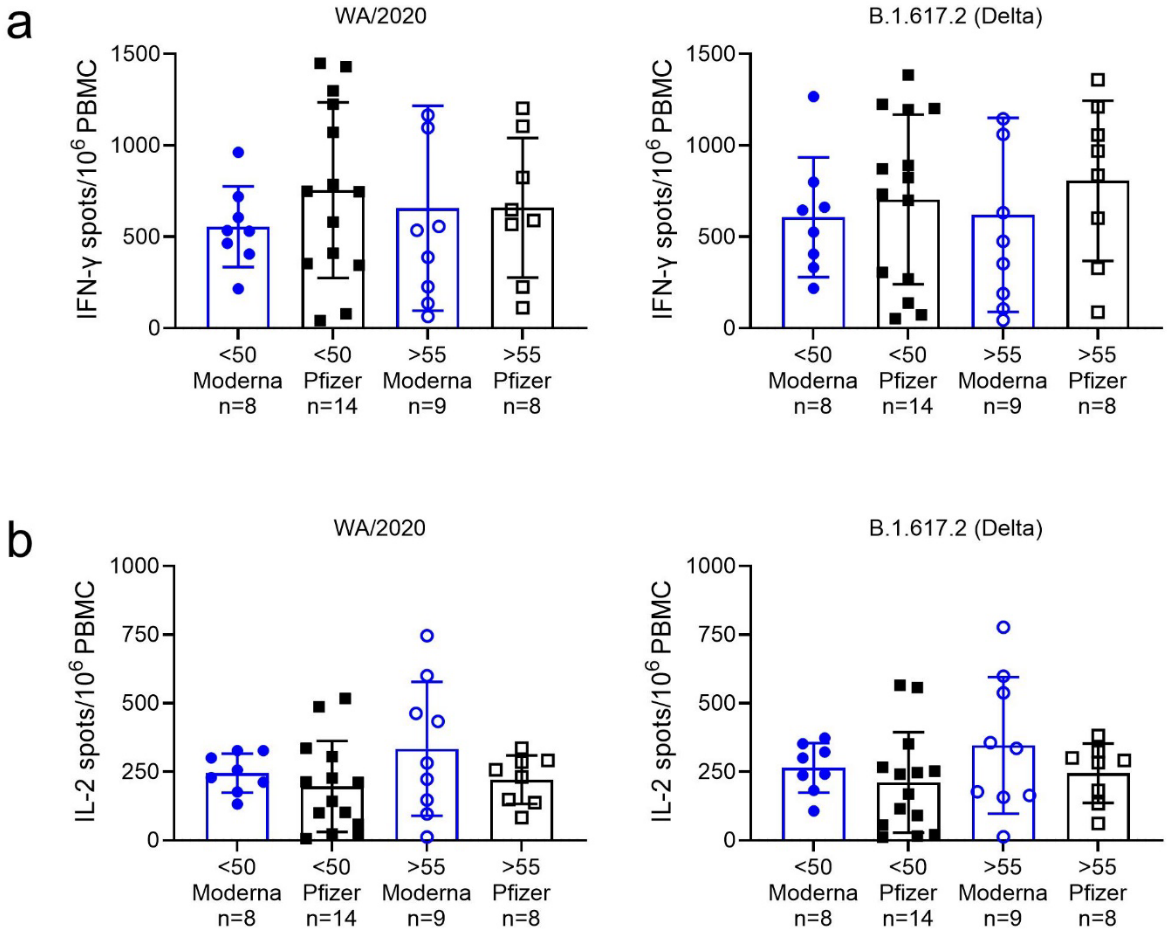

Supplementary Fig 3. Vaccine brand did not differentially impact T cell responses in these cohorts. Number of IFN-γ ELISpots (**a**) or IL-2 ELISpots (**b**) was not statistically different between recipients of Moderna and Pfizer mRNA vaccines at the T4 time point. Kruskal-Wallis test with Dunn's post-hoc correction. All data presented as mean values +/- SEM.

Supplemental Table 1. List of antibodies used for flow cytometry analysis.

Antigen	Fluorochrome	Ab. clone	Manufacturer	Cat.No.	Dilution
CD19	Spark NIR™ 685	HIB19	Biolegend	302270	1/50
CD27	FITC	0323	Biolegend	302806	1/50
CD38	BV650	HB-7	Biolegend	356620	1/50
IgD	BV480	11-26C.1	Becton Dickinson	566138	1/50
IgM	APC	MHM-88	Biolegend	314510	1/50
CD11c	APCe780	3.9	ThermoFisher	47-0116-42	1/25
CD3	BV510	OKT3	Biolegend	317332	1/50
CD21	Pedazzle594	Bu32	Biolegend	354922	1/50
CD4	SparkBlue550	SK3	Biolegend	344656	1/100
CD13	PECy7	WM15	Biolegend	301712	1/50
CD8	BUV395	RPA-T8	Becton Dickinson	563795	1/50
CD137	PE-Cy5	4B4-1	Biolegend	309808	1/50
