# Supplementary material for: Competent immune responses to SARS-CoV-2 variants in older adults following two doses of mRNA vaccination
Source: Nat Commun. 2022 May 24;13:2891. doi: 10.1038/s41467-022-30617-9 (PMC9130515; doi:10.1038/s41467-022-30617-9)
Supplement: Supplementary file 3 — Reporting Summary [file 41467_2022_30617_MOESM3_ESM.pdf]

nature portfolio

Corresponding author(s): Janko Nikolich-Zugich

Last updated by author(s): 4/12/2022

Reporting Summary

Nature Portfolio wishes to improve the reproducibility of the work that we publish. This form provides structure for consistency and transparency in reporting. For further information on Nature Portfolio policies, see our Editorial Policies and the Editorial Policy Checklist.

Statistics

For all statistical analyses, confirm that the following items are present in the figure legend, table legend, main text, or Methods section.

n/a	Confirmed	
☐	☒	The exact sample size (*n*) for each experimental group/condition, given as a discrete number and unit of measurement
☐	☒	A statement on whether measurements were taken from distinct samples or whether the same sample was measured repeatedly
☐	☒	The statistical test(s) used AND whether they are one- or two-sided<br>*Only common tests should be described solely by name; describe more complex techniques in the Methods section.*
☐	☒	A description of all covariates tested
☐	☒	A description of any assumptions or corrections, such as tests of normality and adjustment for multiple comparisons
☐	☒	A full description of the statistical parameters including central tendency (e.g. means) or other basic estimates (e.g. regression coefficient) AND variation (e.g. standard deviation) or associated estimates of uncertainty (e.g. confidence intervals)
☐	☒	For null hypothesis testing, the test statistic (e.g. *F*, *t*, *r*) with confidence intervals, effect sizes, degrees of freedom and *P* value noted<br>*Give P values as exact values whenever suitable.*
☒	☐	For Bayesian analysis, information on the choice of priors and Markov chain Monte Carlo settings
☒	☐	For hierarchical and complex designs, identification of the appropriate level for tests and full reporting of outcomes
☒	☐	Estimates of effect sizes (e.g. Cohen's *d*, Pearson's *r*), indicating how they were calculated

Our web collection on statistics for biologists contains articles on many of the points above.

Software and code

Policy information about availability of computer code

Data collection	CTL mmunoSpot® 7.0 Pro DC<br>Mabtech Apex 1.1<br>SpectroFlo
Data analysis	Flowjo v10<br>Graphpad prism 7<br>CTL mmunoSpot® 7.0 Pro DC<br>Mabtech Apex 1.1

For manuscripts utilizing custom algorithms or software that are central to the research but not yet described in published literature, software must be made available to editors and reviewers. We strongly encourage code deposition in a community repository (e.g. GitHub). See the Nature Portfolio guidelines for submitting code & software for further information.

Data

Policy information about availability of data

All manuscripts must include a data availability statement. This statement should provide the following information, where applicable:

- Accession codes, unique identifiers, or web links for publicly available datasets
- A description of any restrictions on data availability
- For clinical datasets or third party data, please ensure that the statement adheres to our policy

Source data are provided with this paper. The flow cytometric data used for analysis of SARS-CoV-2 specific B cells in Fig. 3 has been deposited to flow repository.org and is accessible through accession number FR-FCM-Z56D.

Field-specific reporting

Please select the one below that is the best fit for your research. If you are not sure, read the appropriate sections before making your selection.

☒ Life sciences ☐ Behavioural & social sciences ☐ Ecological, evolutionary & environmental sciences

For a reference copy of the document with all sections, see nature.com/documents/nr-reporting-summary-flat.pdf

Life sciences study design

All studies must disclose on these points even when the disclosure is negative.

Sample size	Our final cohort included 23 participants under 50 years of age (<50) and 17 participants over the age of 55 (>55). All participants recruited before December 2021 and for which prevaccination bleed and post vaccination bleeds were obtained were recruited in the study.
Data exclusions	One participant was excluded from the study because they had a high neutralizing antibody titer and a strong antigen specific T cell response before vaccination, indicating prior infection.
Replication	No replication was done as human sample is limited.
Randomization	Participants were allocated to study groups exclusively by chronological age.
Blinding	All the subject samples were deidentified and assigned name codes. Researchers were not aware of participant age and group during data collection and analysis.

Reporting for specific materials, systems and methods

We require information from authors about some types of materials, experimental systems and methods used in many studies. Here, indicate whether each material, system or method listed is relevant to your study. If you are not sure if a list item applies to your research, read the appropriate section before selecting a response.

Materials & experimental systems

n/a	Involved in the study	
☐	☒	Antibodies
☐	☒	Eukaryotic cell lines
☒	☐	Palaeontology and archaeology
☒	☐	Animals and other organisms
☐	☒	Human research participants
☒	☐	Clinical data
☒	☐	Dual use research of concern

Methods

n/a	Involved in the study	
☒	☐	ChIP-seq
☐	☒	Flow cytometry
☒	☐	MRI-based neuroimaging

Antibodies

Antibodies used

Marker Fluorochrome Manufacturer Cat.No. Clone
CD19 Spark NIR™ 685 Biolegend 302270
HIB19
CD27 FITC Biolegend 302806 0323
CD38 BV650 Biolegend 356620
HB-7
CD11c APCe780 Thermofisher 47-0116-42 3.9
IgD BV480 BD 566138 11-26C.1
IgM APC Biolegend 314510
MHM-88
CD3 BV510 Biolegend 317332
OKT3
CD21 Pedazzle594 Biolegend 354922
Bu32
CD4 SparkBlue550 Biolegend 344656
SK3
CD13 PECy7 Biolegend 301712
WM15
CD8 BUV395 BD 563795 RPA-T8
CD137 PE-Cy5 Biolegend 309808
4B4-1

	OX-40 PerCPCy5.5 Biolegend 350010 Ber-ACT35
Validation	Antibodies were validated by the manufacturer. For all Biolegend antibodies: Each lot of this antibody is quality control tested by immunofluorescent staining with flow cytometric analysis.<br>For Thermofisher 47-0116-42 3.9: This 3.9 antibody has been pre-titrated and tested by flow cytometric analysis of human peripheral blood cells.

Eukaryotic cell lines

Policy information about cell lines

Cell line source(s)	Vero Cells (ATCC #CCL-81), Calu-3 Cells (ATCC #HTB-55)
Authentication	None of the cell lines were authenticated
Mycoplasma contamination	The cells were confirmed mycoplasma negative by PCR screening.
Commonly misidentified lines<br>(See ICLAC register)	No commonly misidentified lines were used in this study.

Human research participants

Policy information about studies involving human research participants

Population characteristics	Over 21 years old, both genders, any person who was eligible for COVID vaccination
Recruitment	Participants were recruited from the local community by posting a flier in University buildings and social media. Self selection bias likely did not influence results as the study was concerned only with vaccinated individuals.
Ethics oversight	University of Arizona Institutional Review Board (IRB) Protocol number 2102460536

Note that full information on the approval of the study protocol must also be provided in the manuscript.

Flow Cytometry

Plots

Confirm that:

☒ The axis labels state the marker and fluorochrome used (e.g. CD4-FITC).

☒ The axis scales are clearly visible. Include numbers along axes only for bottom left plot of group (a 'group' is an analysis of identical markers).

☒ All plots are contour plots with outliers or pseudocolor plots.

☒ A numerical value for number of cells or percentage (with statistics) is provided.

Methodology

Sample preparation	Cryopreserved PBMC (2-5 x 106/sample) were thawed in prewarmed RPMI-1640 with L-glutamine (Lonza) + 10% FCS. Thawed PBMCS were rested overnight at 37 °C in X-VIVO 15 Serum-free Hematopoietic Cell Medium (Lonza) supplemented with 5% human Ab serum. Cells were stained with surface antibodies in PBS (Lonza) + 2% FCS, and 307 then stained with the live dead fixable blue dye (Thermofisher).
Instrument	Cytek Aurora
Software	FCS files were analyzed by FlowJo software (Tree Star).
Cell population abundance	At least 10000 CD19+ lymphocytes were recorded for each participant.
Gating strategy	Lymphocytes were identified by size and granularity in the FSC/SSC scatter. Doublets were excluded by FSC width and area.Dead cells were excluded using live/dead fixable dyes.

☒ Tick this box to confirm that a figure exemplifying the gating strategy is provided in the Supplementary Information.
